# Rheumatoid meningitis developed in patient with stable rheumatoid arthritis and myasthenia gravis—detailed analysis of intracranial inflammation using flow cytometry

**DOI:** 10.1186/s12974-018-1196-3

**Published:** 2018-05-18

**Authors:** Miki Oono, Yoshimasa Fujita, Nobuaki Uchida, Ukichiro Kawai, Michiyo Fujita-Nakata, Megumi Nakanishi, Mitsuru Sanada, Shigemi Nagayama, Makoto Matsui

**Affiliations:** 1 Department of Neurology, Kanazawa Medical University, 1-1 Daigaku, Uchinada, Kahoku-gun, Ishikawa 920-0293 Japan; 2 Department of Hematology and Immunology, Kanazawa Medical University, 1-1 Daigaku, Uchinada, Kahoku-gun, Ishikawa 920-0293 Japan

**Keywords:** Rheumatoid meningitis, Myasthenia gravis, Rheumatoid arthritis, Flow cytometry, Cerebrospinal fluid, Interleukin-6, Humoral immunity

## Abstract

**Background:**

Rheumatoid meningitis (RM) is a rare disorder that often develops during a remission phase of rheumatoid arthritis (RA). This is the first study to demonstrate differences in regard to immunological disturbance between blood and cerebrospinal fluid (CSF) samples obtained from a patient with RM using flow cytometry.

**Case presentation:**

A 36-year-old woman with RA and generalized myasthenia gravis (MG) developed RM during a remission phase. Although both RA and MG were stable and well controlled, she noticed fever, headache, and transient sensory disturbance. Blood and CSF examination findings suggested aseptic meningitis, while brain magnetic resonance imaging revealed restricted portions of meningitis and associated cortical lesions, compatible with a diagnosis of RM. The dose of oral prednisolone was increased, which ameliorated the symptoms within 1 week along with improvement in CSF findings. This patient exhibited features of RM that were manifested in a manner independent of the activity of RA. An investigation of cellular immunity using CSF specimens with flow cytometry showed differences in regard to the pathogenesis of inflammation in the CSF as compared to outside of the central nervous system. In contrast to results obtained with paired blood samples, CSF cells at the peak stage of RM showed a marked increase in CCR3^+^ Th2 cells and marked decrease in CD8^+^ cells, suggesting an immunoregulatory disturbance in the CSF. Those findings indicated a CSF-specific activation of humoral immunity, resulting in augmentation of meningeal inflammation, as shown by excess synthesis of intrathecal IgG and markedly elevated interleukin-6 level. Results of the present detailed investigation of lymphocyte subsets revealed a discrepancy regarding the process of inflammation in this RM patient between CSF and blood samples.

**Conclusions:**

RM is not a simple reflection of the immune status of RA, as the pathogenesis seems related to, at least in part, CSF-specific immunological dysregulation.

## Background

Development of rheumatoid meningitis (RM), a rare autoimmune inflammatory disease of the central nervous system (CNS), is often seen in well-controlled rheumatoid arthritis (RA) patients [1, 2] or even manifested as the first symptom in patients with RA [3, 4]. We treated a young woman diagnosed with myasthenia gravis (MG) and RA, who presented with meningitis and transient sensory disturbance during a remission phase of both disorders. A flow cytometry investigation of cellular immunity using cerebrospinal fluid (CSF) and blood samples showed differences in regard to the pathogenesis of inflammation in the CSF and outside of the CNS.

## Case presentation

A 36-year-old woman with a 13-year history of generalized MG was diagnosed with RA 8 years prior to the present episode. The patient had undergone a thymectomy, and both diseases were stable with alternate-day administrations of prednisolone (8 mg) and weekly doses of methotrexate (12 mg) until she exhibited new neurological complications. At the time of the initial visit, the patient reported a 10-day history of headache in the left parietal region and also had a moderate fever of 38 °C. On the sixth day after developing the headache, a transient sensory disturbance (dysesthesia) over the right face developed and then spread to the right upper extremity for 30 min, which recurred twice that day; thus, she came to us and also noted headache and fever but no pain in any joints. Neurological examination findings were negative for meningeal irritation signs, and there were no symptoms of MG including ptosis, diplopia, dysarthria, or weakness in the four extremities. Mental state, deep tendon reflexes, and coordination were normal, and there was no sensory disturbance. The patient had no past history of hypertension, diabetes, or smoking.

**Fig. 1:**
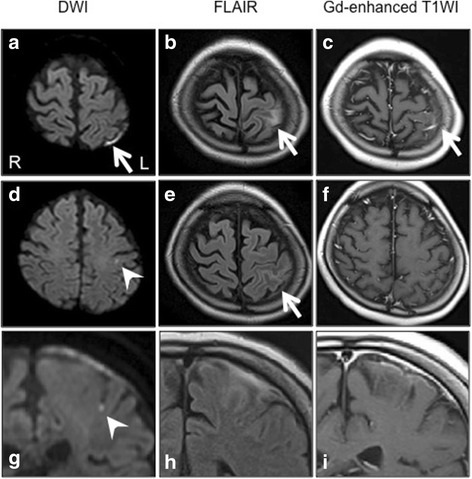
Brain MRI scan findings at admission. **a** Axial DWI showing restriction of diffusion in the left temporal subarachnoid space (arrow). **b**, **e** Axial FLAIR images showing hyperintensity from the same lesion. **c**, **f** Axial Gd-enhanced T1-weighted images showing partial enhancement in the left temporal subarachnoid space. **d** Axial DWI showing a spot lesion indicating restriction of diffusion in the left parietal cortex (arrowhead). **g**–**i** Coronal-DWI, FLAIR, and Gd-enhanced T1-weighted views of the same lesion shown in **d**, **e**, and **f**. DWI diffusion-weighted imaging, FLAIR fluid-attenuated inversion recovery, Gd gadolinium

Routine laboratory tests revealed elevated C-reactive protein (CRP) (6.09 mg/dl, normal < 0.3 mg/dl) and erythrocyte sedimentation rate (ESR) (56 mm in 1 h, normal < 3–11 mm), while white blood cells were normal (7980/μl, normal 3040–8720/ μl; neutrophils, 76.8%, normal 40–77%; lymphocytes, 11.6%, normal 16–44%). Serum electrolytes, creatine, liver enzymes, and the coagulation system were also normal, while titers for anti-acetylcholine receptor (AChR) (12 nmol/l, normal < 0.2 nmol/l) and anti-ribonucleoprotein (RNP) (15 U/ml, normal < 0.5 U/ml) antibodies were elevated, though to the same extent as seen in previous examinations. Complements, as well as cytoplasmic and perinuclear anti-neutrophil cytoplasmic antibodies, were normal. The CSF sample obtained at admission was lymphocytic (white cell count 19/μl, normal < 5, predominance of mononuclear cells) with elevated levels of protein (57 mg/dl, normal 15–45 mg/dl) and IgG (7.0 mg/dl, normal 0.5–4.0 mg/dl; IgG index 0.80, normal < 0.7) and a normal glucose level (51 mg/dl, normal 50–80; serum glucose 116 mg/dl; CSF:serum ratio 0.81). The oligoclonal IgG band was positive (seven bands), and intrathecal IgG synthesis was elevated (6.0 mg/day, normal < 3.3 according to previous report [5]). Furthermore, interleukin-6 (IL-6) was markedly elevated in the CSF (843 pg/ml, normal < 12.1 pg/ml according to previous report [6]). Other CSF examination results were negative, including polymerase chain reaction analysis for infectious agents (herpes simplex, varicella zoster, cytomegalovirus, tuberculosis), cultures for bacteria and acid-fast bacilli, India ink capsule staining, and cytology for malignant cells. Brain magnetic resonance imaging (MRI) using fluid-attenuated inversion recovery (FLAIR) and diffusion-weighted imaging (DWI) showed hyperintense lesions in the subarachnoid space over the left parietal lobe and cortex adjacent to the meningeal lesion (Fig. 1a, b). The meningeal lesion was enhanced by gadolinium (Gd) (Fig. 1c). Moreover, DWI revealed a spot lesion suggesting the presence of an ischemic lesion in the left parietal cortex (Fig. 1d). No abnormalities were seen in magnetic resonance angiogram findings.

### Flow cytometric analysis

Flow cytometry examinations of blood and CSF samples were performed prior to giving additional treatment for RM, using previously described methods [7]. The CSF sample obtained at admission was characterized by a marked increase in clusters of differentiation (CD) 4-positive helper T cells and a decrease in CD8-positive T cells, with a markedly elevated CD4/8 ratio of 11.5, whereas the blood sample showed only a moderate increase in B cells and normal CD4/8 ratio of 2.5 (Table 1). C-C chemokine receptor type 5 (CCR5) and C-X-C chemokine receptor type 3 (CXCR3) are characteristic molecules expressed by type 1 helper T (Th1) cells, while CCR3 and CCR4 are expressed by Th2 cells [8]. Furthermore, the CD29 antigen has been shown effective to define helper-inducer T cells [9]. We used monoclonal antibodies against those surface molecules to identify helper T cell subsets capable of propagating immune reaction. Our findings revealed that the same CSF sample showed a marked increase in CCR3-positive Th2 cells in contrast to a modest increase of such cells in paired blood samples shown in results obtained in our previous study of viral meningitis (Table 1) [10].

**Table 1:** Lymphocyte subsets in blood and CSF samples obtained prior to treatment for rheumatoid meningitis

Lymphocyte subset	Function	Blood (%)	Viral meningitis	CSF (%)	Viral meningitis
CD3	Mature T cell	55.6	75.0 ± 7.4	88.8	86.7 ± 8.7
CD4	Helper T cell	40.2	41.4 ± 11.1	81.5	66.3 ± 10.2
CD8	Suppressor/cytotoxic T cell	16.2	33.0 ± 14.3	7.1	21.6 ± 8.4
CD19	B cell	26.7	9.2 ± 3.8	3.8	3.7 ± 6.5
CD3-CD16+CD56+	NK cell	18.9	13.9 ± 6.6	5.3	6.3 ± 4.1
CD4+CD29+	Helper-inducer T cell	19.8	17.7 ± 4.8	69.2	47.7 ± 12.2
CD4+CCR5+	Th1 cell	2.4	3.2 ± 1.6	21.4	16.3 ± 13.3
CD4+CXCR3+	Th1 cell	8.7	11.7 ± 3.7	37.6	41.3 ± 13.0
CD4+CCR3+	Th2 cell	11.4	1.4 ± 1.7	38.3	8.5 ± 16.4
CD4+CCR4+	Th2 cell	1.3	3.1 ± 2.5	10.4	10.4 ± 15.2
CD4/8		2.50		11.50	

Values for viral meningitis were adopted from the results of a previous study, designated as [10]

*CSF* cerebrospinal fluid, *NK* natural killer, *Th1* type 1 helper T, *Th2* type 2 helper T

### Treatment and outcome

**Fig. 2:**
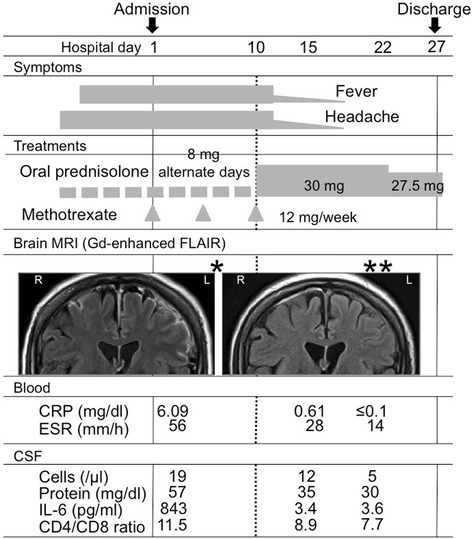
Clinical course of the patient. Fever and headache rapidly improved after starting administration of oral prednisolone (30 mg/day). Coronal section images obtained with brain MRI and Gd-enhanced FLAIR revealed high intensity in the subarachnoid space in the bilateral parietal lobes (left dominant) (single asterisk). At 12 days after starting treatment, lesion size was reduced and high intensity in the cortex was decreased (double asterisks)

On the basis of laboratory and MRI findings, a diagnosis of RM was made and oral prednisolone administration was increased to a total dose of 30 mg/day, while that of methotrexate was discontinued. Two days later, the fever and headache were ameliorated, followed by improvements in laboratory data, including CRP, ESR, and CSF cell count (12/μl), as well as levels of protein (35 mg/dl) and IL-6 (3.4 pg/ml) in the CSF (Fig. 2). After 3 months, the dose of oral prednisolone was tapered to 15 mg/day and weekly methotrexate at 8 mg was re-started. Brain MRI findings remained for 7 months, though without manifestation of neurological symptoms or signs.

## Discussion and conclusions

We treated a patient with RM who had a long history of MG and RA. This case showed reported features of RM, which were manifested independently of RA activity [1, 2], as well as neurological symptoms, including transient sensory disturbance [2, 11, 12]. Although most reported cases of RM include brain/meningeal biopsy findings, asymmetrical meningeal involvement revealed by MRI [1, 2, 6, 11–13] has been found helpful for an early diagnosis of RM, which contributes to improvement of treatment outcome [13, 14]. Indeed, some recent cases were diagnosed without biopsy findings, including the present [3, 6, 15]. The presence of vasculitis was reported in a biopsy specimen obtained from a case of RM [13], and cortical lesions likely develop as a consequence of impaired blood flow beneath the restricted area of inflammatory meningeal lesions. It is also conceivable that minor damage or ischemia in the cortex is manifested as transient ischemic attacks or seizure-like episodes. Furthermore, the fever noted in the present patient deserves mention, as this symptom has been reported only rarely even when the feeling of chills is included [6, 12, 14]. Interestingly, a previously reported patient with a fever of 38.2 °C [6] showed extremely high levels of IL-6 in the CSF the same as the present patient, suggesting an ongoing intense inflammatory process in the CSF/meninges.

In the present case, it is noteworthy that RM developed during a phase of remission of both RA and generalized MG. If an extrinsic factor is present, such as an infectious event that can induce general T and/or B cell activation before development of RM, symptoms related to RA and MG will likely worsen along with elevation of immunological markers for those disorders. The good response of clinical symptoms, as well as improvement in ESR and CRP following administration of a moderate dose of corticosteroids alone in the present case and others [4], is similar to polymyalgia rheumatica. However, in our patient, no painful symptoms except for headache were noted during the entire course of illness. Instead, we found a marked increase in the CD4-positive helper T cell population, especially CCR3-positive Th2 cells, as well as marked decrease in CD8-positive cells in the CSF, in contrast to modest changes in those cells in blood. A subset of CD8-positive cells is known to have a role as suppressor T cells [16]; thus, we concluded that immunoregulation in the CSF was disturbed in the present case. Although B cells were increased along with a relative decrease in CD3-positive mature T cells in blood as compared to the results of our previous study of viral meningitis and non-inflammatory neurological diseases [10], these findings may have been due to the effects of long-term immunosuppressive therapy with methotrexate. Furthermore, CSF samples showed an elevated IgG index, positive oligoclonal bands, and excessive intrathecal IgG synthesis. In light of the marked increase in Th2 cells in the CSF, these results indicate the presence of CSF-specific activation of humoral immunity resulting in propagation of meningeal inflammation, though the triggering factor or precise mechanism was not addressed in the present study. Another limitation is that we did not measure the level of IgG4 in serum. However, IgG4-related disease seems to be less likely in cases of asymmetric leptomeningitis [13].

In summary, we performed flow cytometric analysis of cellular immunity in a patient with RM, which showed that immunoregulatory disturbance associated with elevated Th2-type response in the CSF may have stimulated intrathecal IgG synthesis. Results obtained in this case indicate that the pathogenesis of RM differs from that of a systemic manifestation of RA and MG.

## Abbreviations

AChR: Acetylcholine receptor
ANCA: Anti-neutrophil cytoplasmic antibodies
CCR: C-C chemokine receptor
CD: Cluster of differentiation
CRP: C-reactive protein
CSF: Cerebrospinal fluid
DWI: Diffusion-weighted imaging
ESR: Erythrocyte sedimentation rate
FLAIR: Fluid-attenuated inversion recovery
Gd: Gadolinium
IL-6: Interleukin-6
MG: Myasthenia gravis
MRI: Magnetic resonance imaging
NK: Natural killer
RA: Rheumatoid arthritis
RM: Rheumatoid meningitis
RNP: Ribonucleoprotein
Th2: Type 2 helper T
TIA: Transient ischemic attack
